# Prediction and analysis of components and functions of *Ixeris chinensis* based on network pharmacology and molecular docking

**DOI:** 10.3389/fmed.2024.1360966

**Published:** 2024-06-27

**Authors:** Ziwei Ni, Zhe Ma, Xiaoting Qiao, Yaqian Guo, Cailian Ruan, Yayun Wang, Ying Yang

**Affiliations:** ^1^Medical School of Yan’an University, Yan'an, Shaanxi, China; ^2^Ultrasound Room of Shaanxi Provincial Hospital of Traditional Chinese Medicine, Xi'an, Shaanxi, China; ^3^Xi'an Jiaotong University School of Medicine, Xi'an, Shaanxi, China; ^4^National Experimental Center of Air Force Medical University, Xi'an, Shaanxi, China; ^5^Xi'an Children's Hospital Research Institute, Xi'an, Shaanxi, China

**Keywords:** *Ixeris chinensis*, network pharmacology, molecular docking, meta analysis, drug development

## Abstract

**Background:**

It is reported that the *Ixeris chinensis* has high medicinal value, but there are few reports about its potential molecular mechanism. We used a network pharmacology approach to predict the active ingredients, targets of action and possible interventions in diseases of *Ixeris chinensis*.

**Methods:**

We employed various databases and software to predict the active ingredients, target genes, protein interactions, signaling pathways, network diagrams, and molecular docking of *Ixeris chinensis*. Simultaneously, we searched multiple Chinese and English databases and conducted meta-analyses of five randomized controlled trials.

**Results:**

The analysis results revealed 12 effective components, including apigenin *β*-sitosterol, baicalin, baicalein, and luteolin; and selected 40 key targets, including AKT1, TNF, EGFR, ESR1, SRC, among others. GO analysis generated 225 biological processes, 39 cellular components, and 65 molecular functions; KEGG analysis revealed 103 signaling pathways. Molecular docking results indicated that the main active components of *Ixeris chinensis* can bind well with key targets. Five randomized controlled trials were included. Meta-analysis showed that Ixeris extract can effectively reduce animal blood lipid levels.

**Conclusion:**

This study revealed the main active ingredients and key targets of *Ixeris chinensis*, analyzed the signaling pathways of potential targets, conducted disease prediction, and performed molecular docking prediction, providing a basis for research on the pathways of Ixeris treatment for related diseases and subsequent new drug development.

## 1 Introduction

*Ixeris chinensis (Thunb.)* Nakai is the whole herb of *Sonchus* of the family Asteraceae (1). According to the textual research on the herbal literature, the authentic source of Chinese herbal medicine bitter vegetables should be the main stream of *Sonchus oleraceus L*, which belongs to *Sonchus*, *Ixeris polycephala* Cass of Ixeris Cass, and the dried whole grass of Chinese Ixeris (2). *I. chinensis* is a traditional medicinal plant, which can be used to treat tumors, inflammation and some infections. *I. chinensis* contains a variety of flavonoids, which have various pharmacological or health effects such as antioxidant, anti-cancer, antihypertensive, improving cardiovascular diseases, lowering blood lipids, lowering cholesterol, and protecting our liver (3). *I. chinensis* is one of the traditional Chinese herbs in China, with a wide range of distribution, easy survival and a wide variety of species (4).

In recent years, with the deepening of the research on *I. chinensis*, the clinical application scope of the herb has been continuously expanded (5). It is often used in combination with other Chinese medicines for treating various inflammatory diseases such as gastritis, enteritis, lung fever and cough, sore throat, etc. (6). The pharmacological effects of *I. chinensis* have been studied more extensively, but the mechanism of action of the disease is still unknown and needs to be studied thoroughly.

Network pharmacology research uses public databases and literature to screen out the target information of known drug components (7). Then uses network visualization tools to build a multi-dimensional biological network model, through multi-level analysis of specific signal nodes in the network at the cellular, molecular and overall biological levels, to identify key nodes, and from the perspective of the overall biological network balance to discover the target drug’s interference with the “pathogenic network,” and then predicts the pharmacological active ingredients, targets and possible involvement in the regulation of cellular signal transduction pathways, potential pharmacological mechanisms and formulation and combination patterns of known drugs to combat diseases (8). To predict the active ingredients, targets, cellular signal transduction pathways, potential pharmacological mechanisms and prescriptions that may be involved in the regulation of known drugs against diseases (9). Finally, validation at the animal or cellular level, will reveal the modern pharmacological mechanisms of drugs against diseases and explore new indications for drug interventions (10).

This artical adopted the research method of network pharmacology. By screening the active components and key targets of *I. chinensis*, we systematically analyzed its potential action targets to construct protein interaction networks and the enriched biological processes and pathways, so as to elaborate the pharmacological action mechanism of *I. chinensis*, which laid a foundation for further in-depth study on the mechanism of action of *I. chinensis* in the treatment of specific diseases and was of great significance in the field of new drug development.

## 2 Materials and methods

### 2.1 Database

Traditional Chinese Medicine Systems Pharmacology (TCMSP^1^), HERB database^2^, Pubchem database^3^, Swiss Target Prediction database^4^, Uniprot database^5^, STRING database^6^, DAVID database^7^, pubmed^8^, Web of Science^9^, China National Knowledge Infrastructure (CNKI^10^), Wangfang database^11^.

### 2.2 Composition software

Cytoscape3.9.1, Rstudio, Microlife Letter, PyMOL, AutoDockTools, RevMan 5.4.1.

### 2.3 Screening of monomeric active compounds in Chinese medicine

By searching the Traditional Chinese Medicine Systems Pharmacology Database and Analysis Platform. The results were obtained by searching for “*Sonchus oleraceus* L,” “*Ixeris polycephala* Cass,” “*I. chinensis* (Thunb.) Nakai,” “*Lactuca tatarica*.” The active ingredients were searched in the HERB database by searching for “*I. chinensis.”*

### 2.4 Target screening of active compounds

We inputted the active components of *I. chinensis* into the swiss target prediction to predict the target genes, and used the Uniprot database (see foot note 5) to convert the full name of the target gene to the abbreviation, deleted the target without corresponding gene name, and used Cytoscape 3.9.1 to construct the network diagram of *I. chinensis* – component-target.

### 2.5 Critical targets screening

The potential targets were entered into the STRING database for protein interaction analysis, free targets were excluded, and the Protein–protein interaction (PPI) network was constructed using Cytoscape 3.9.1 software to screen for key targets, which could be considered as important proteins for bittercress to exert its medicinal effects.

### 2.6 Enrichment analysis

We used the DAVID database to perform Gene Ontology (GO) analysis and Kyoto Encyclopedia of Genes and Genomes (KEGG) pathway enrichment analysis on key targets, including Biological Process (BP), Cell Component (CC) and Molecular Function (MF). With human gene as the background, set *p-*value<0.01, we screened the top 10 BP, CC, MF entries and the top 20 KEGG pathway entries, and used the microbiota website to make enrichment histogram and bubble chart.

### 2.7 Prediction of target diseases

We used the Rstudio software to analyze DO data, and used the microbiota website for visual analysis. According to the *p-*value, we screened the top 20 target diseases with the highest credibility, so as to achieve the effect of disease prediction.

### 2.8 Molecular docking technology

According to the PPI analysis results, we selected the top ten key targets AKT1, TNF, EGFR, ESR1, SRC, PTGS2, MAPK1, MMP9, IL2 and AR for docking. Based on the comprehensive analysis of the results of “component-target network” and “component- target-disease network,” the key components in *I. chinensis* luteolin, apigenin, chinensiolide c, chinensiolide b, chinensiolide a, *β*-sitosterol, luteolin-7-*O*-*β*-D-glucoside played a leading role in the network. We adopted semi flexible docking to find the best binding position and strength of substrate molecules and receptor molecules (11). We searched the 3D structure of key proteins in the PDB protein structure database, and selected the proteins with high resolution and long structure in the human background. PyMOL software was used to remove water and solvent molecules in the protein, and AutoDockTools 1.5.7 software was used to conduct hydrogenation treatment, set as ligand. After downloading the 3D structure of small molecules using Pubchem, swiss target prediction, hydrogenation was performed in AutoDockTools 1.5.7 software to automatically assign charges, set up as ligands and set up torsional bonds. We docked large and small molecules, set docking boxes, docked parameters and arithmetic methods. The screened macromolecules and small molecules from *I. chinensis* were docked separately, and the binding energy less than 0 indicated that the ligand and the receptor could bind spontaneously, the binding energy ≤ −5. 0 kcal·mol ^−1^ proved that the component was well docked with the target site, and the binding energy ≤ −7. 0 kcal·mol ^−1^ indicated that the component and the binding conformation of the target was strongly active (12). The docking results were thermographed using Rstudio, and those with high binding energy were visualized using PyMOL.

### 2.9 Meta analysis

#### 2.9.1 Research type

Randomized controlled trial (RCT) of *I. chinensis* water extract in the treatment of diseases.

#### 2.9.2 Research objects

Mice fed with high-fat diet or injected with ccl4 and successfully modeled.

#### 2.9.3 Intervention measures

Test group treated rats with *I. chinensis* water extract. The control group used placebo or no special treatment.

#### 2.9.4 Outcome indicators

Total Cholesterol (TC), Triglycerides (TG), High-Density Lipoprotein Cholesterol (HDL-C), Low-Density Lipoprotein Cholesterol (LDL-C), Malondialdehyde (MDA).

#### 2.9.5 Literature retrieval strategy

Subject term retrieval (see footnote 8), Web of Science (see footnote 9), CNKI (see footnote 10), Wanfang database (Wanfang, see footnote 11), to be included in the randomized controlled trial with *I. chinensis* as the treatment method. Take Pubmed as an example. See the table for its specific retrieval strategy (Table 1).

**Table 1:** Pubmed retrieval strategy.

#1 TS = ((“asteraceae”[MeSH Terms] OR “asteraceae”[All Fields] OR “ixeris”[All Fields]) AND chinensis[All Fields]) AND ((“disease”[MeSH Terms] OR “disease”[All Fields] OR “diseases”[All Fields]) OR (“disease”[MeSH Terms] OR “disease”[All Fields]))
#2 TS = ((“asteraceae”[MeSH Terms] OR “asteraceae”[All Fields] OR “ixeris”[All Fields]) AND chinensis[All Fields]) AND ((“disease”[MeSH Terms] OR “disease”[All Fields] OR “diseases”[All Fields]) OR (“disease”[MeSH Terms] OR “disease”[All Fields]))
#3 TS = ((“asteraceae”[MeSH Terms] OR “asteraceae”[All Fields] OR “ixeris”[All Fields]) AND chinensis[All Fields]) AND ((“inflammation”[MeSH Terms] OR “inflammation”[All Fields]) OR (“inflammation”[MeSH Terms] OR “inflammation”[All Fields] OR (“innate”[All Fields] AND “inflammatory”[All Fields] AND “response”[All Fields]) OR “innate inflammatory response”[All Fields]) OR (Inflammatory[All Fields] AND Response[All Fields]) OR Innate[All Fields] OR (“inflammation”[MeSH Terms] OR “inflammation”[All Fields] OR (“innate”[All Fields] AND “inflammatory”[All Fields] AND “responses”[All Fields]) OR “innate inflammatory responses”[All Fields]))

#### 2.9.6 For literature quality evaluation

Cochrane risk bias assessment tool and SYRCLE bias risk assessment tool were used to evaluate the quality of the included studies. The evaluation contents included: ① Random sequence generation (selection bias) ② Allocation consideration (selection bias), ③ Blinding of participants and persons (performance bias), ④ Blinding of outcome assessment (detection bias), ⑤ Incomplete outcome data (attribute bias), ⑥ Selective reporting (reporting bias), ⑦ Other biases.

#### 2.9.7 Statistical analysis

RevMan 5.4.1 software was used for meta analysis. The standardized mean difference (SMD) was used as the effect index for the measurement data. The point estimates and 95% CI were given for each effect. If *p* ≥ 0.1 and *I*^2^ < 50%, it was considered that the heterogeneity between the studies was not significant, and the fixed effect model was selected; If *p* < 0.1, *I*^2^ ≥ 50%, it was considered that there was significant heterogeneity among the studies, and the random effect model was selected (13).

## 3 Results

### 3.1 Screening for active compounds of *I. chinensis*

We searched the HERB database for “*I. chinensi*” and found that there were 12 active ingredients, namely Apigenin, *β*-sitosterol, calcium carbonate, chinensiolide a, chinensiolide b, chinensiolide c, ixerisosidea, ixerochinolide, ixerochinoside, Lactucin, Luteolin, luteolin-7-*O*-*β*-D-glucoside. The above active ingredients were used as research subjects.

### 3.2 Analysis of *I. chinensis*–active compound–target network

**Figure 1:**
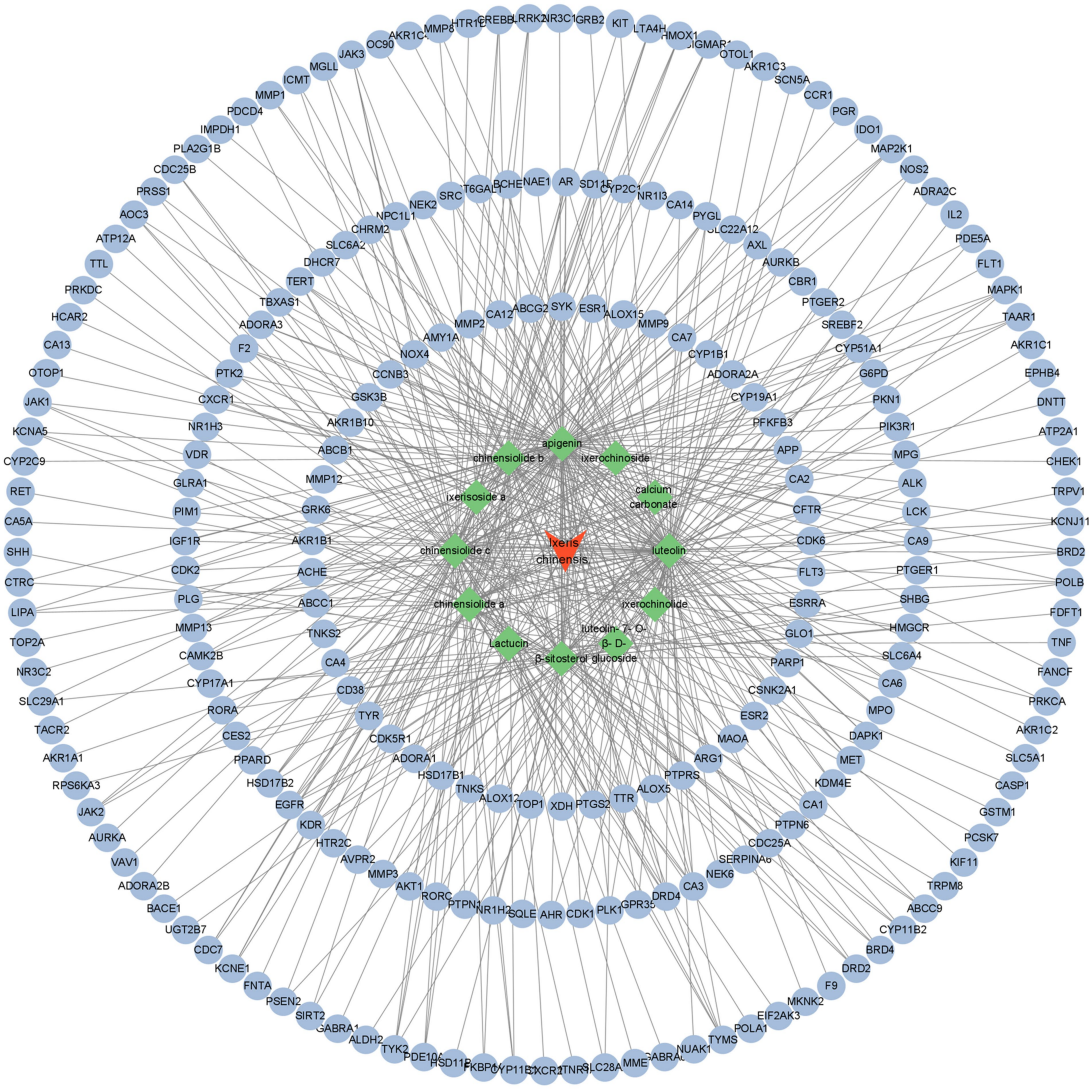
*Ixeris chinensis* active ingredient-target network diagram. The diamond shape represented the active ingredients of this herb, the oval represented the target site.

Since there was no match between the targets of HERB database and the active ingredients of *I. chinensis*, so we entered into Pubchem for searching, and the smileIDs of 10 active ingredients were obtained and then entered into Swiss Target Prediction for target gene prediction, among which the remaining two ingredients, “Lactucin” was used for target gene prediction in TCMSP website and “calcium carbonate” was used for target gene prediction in National Library of Medicine website. By screening the target genes with confidence greater than 0.01 or the top 15 target classes, converting the full names of target genes to abbreviations with the help of Uniprot database (see footnote 5) and deleting the targets without corresponding gene names, a total of 238 targets were derived, and using Cytoscape 3.9.1 to construct the *I. chinensis*–active compound–target network (Figure 1).

### 3.3 Key targets prediction

**Figure 2:**
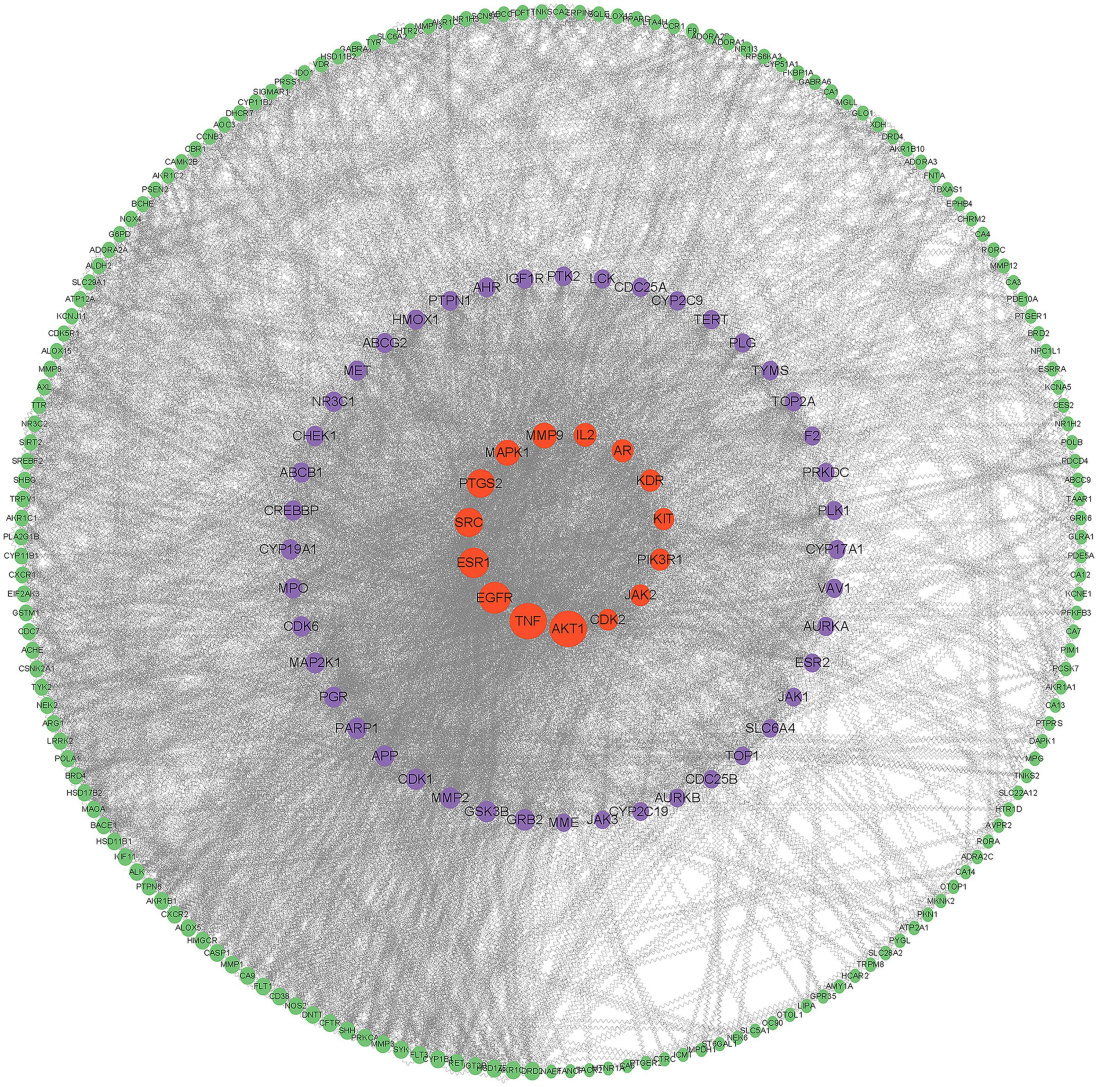
PPI network diagram. The larger the node shape in the figure and the closer to the center, the higher the criticality.

After entering 238 potential targets into STRING database for protein interaction analysis and eliminating free targets, we used Cytoscape 3.9.1 software to construct PPI network (Figure 2) and calculated parameter thresholds, screened Degree (node centrality, reflecting the number of other targets that participate in different disease pathological processes together with a certain target) greater than 30, BC (mediator centrality value, the target with high proximity centrality was directly connected to more targets, indicating the importance of the target in different disease pathomechanical processes) was greater than 369, CC (closeness centrality, reflecting the proximity between a node and other nodes in the network) was greater than 0.0017, a total of 40 key targets were screened, which could be regarded as important proteins for *I. chinensis* to exert their medicinal effects (Table 2).

**Table 2:** Key targets prediction.

Gene	DC	BC	CC
AKT1	194	7225.132999	0.00255102
TNF	192	7903.35269	0.002557545
EGFR	154	3971.227254	0.002409639
ESR1	142	4009.212674	0.00234192
SRC	130	2895.883562	0.002325581
PTGS2	126	3198.407973	0.002293578
MAPK1	104	1673.561859	0.002150538
MMP9	102	1417.735444	0.002136752
IL2	86	595.0008977	0.002061856
AR	80	1267.538745	0.002118644
KIT	72	525.9431779	0.00203666
CDK2	68	709.8494537	0.002012072
GSK3B	66	423.3720516	0.00203666
APP	64	1312.657413	0.002057613
PARP1	64	725.8641357	0.001976285
CDK1	64	463.6579469	0.001941748
PGR	62	591.4637203	0.002074689
CYP19A1	58	1020.976118	0.002
CDK6	58	376.8362946	0.001915709
ABCB1	56	1720.684138	0.002114165
NR3C1	56	939.3539641	0.002053388
CREBBP	56	620.1867078	0.00203252
CHEK1	56	898.8009262	0.001915709
ABCG2	54	1410.390052	0.002020202
AHR	52	909.7985089	0.002004008
CYP2C9	48	638.5874397	0.001964637
TYMS	48	907.4959802	0.001886792
TOP2A	48	524.5084321	0.001808318
F2	46	790.2361428	0.001953125
CYP17A1	46	439.2279507	0.001769912
TOP1	42	381.8070735	0.001901141
SLC6A4	42	1335.630618	0.001883239
CYP2C19	42	622.4386451	0.001841621
DRD2	40	2328.251479	0.001945525
MME	40	682.4157568	0.001883239
AKR1B1	34	1114.208445	0.001937984
CFTR	34	1748.372878	0.001893939
CA9	34	762.4022156	0.001886792
HMGCR	34	633.4694013	0.001865672
HSD11B1	32	376.5306058	0.001782531

### 3.4 GO enrichment analysis of targets

**Figure 3:**
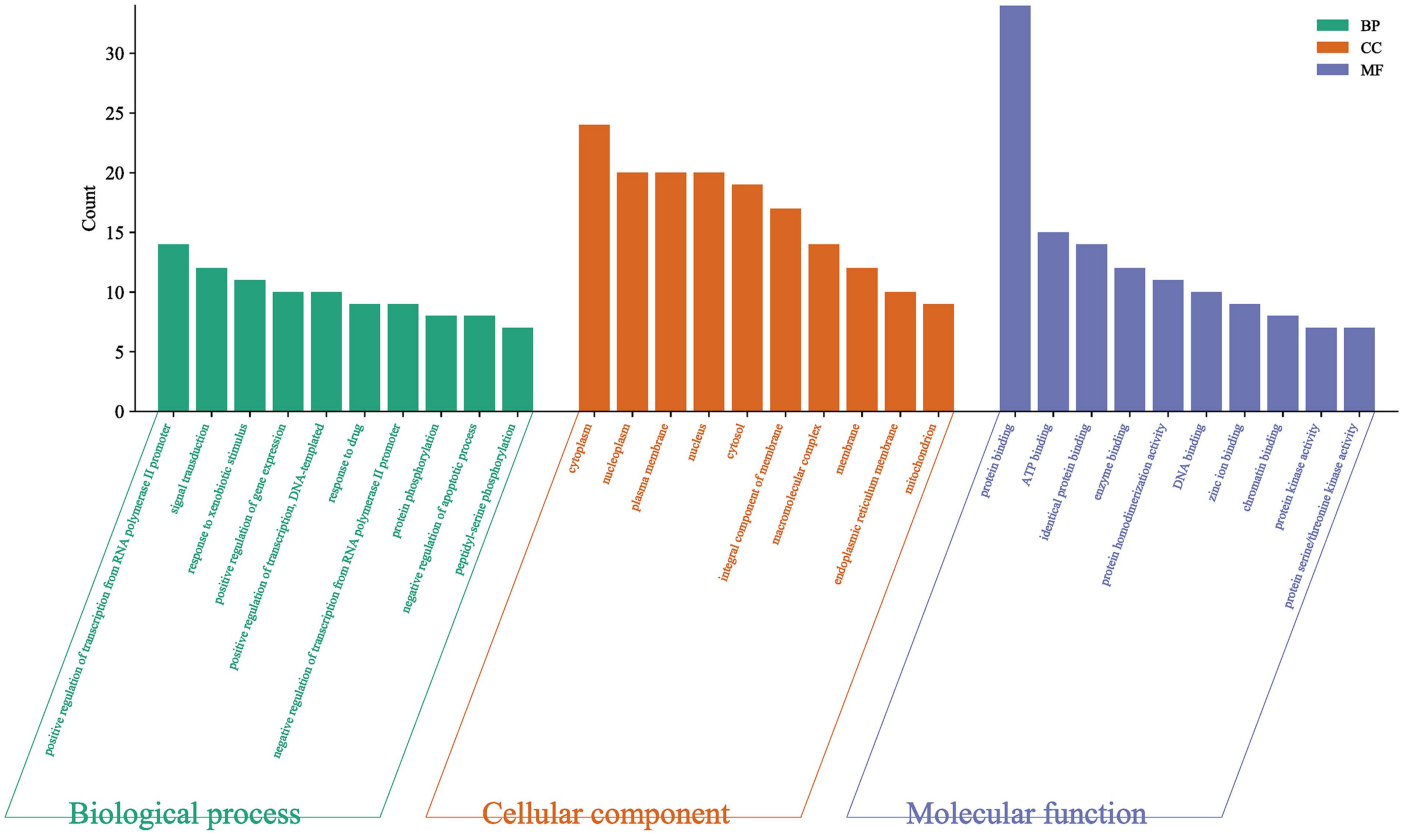
GO enrichment analysis results of key target proteins. Including biological process, cellular components, molecular functions.

GO-BP analysis showed that 225 biological processes were related to this, and the most significant top 5 were positive regulation of transcription from RNA polymerase II promoter, signal transduction, response to xenobiotic stimulus, positive regulation of gene expression, positive regulation of transcription, DNA-templated (Table 3). GO-CC analysis revealed 39 cellular compositions associated with this, the five most significant being cytoplasm, nucleoplasm, plasma membrane, nucleus, cytosol (Table 4). GO-MF analysis showed that 65 molecular functions were related to this, and the most significant 5 were protein binding, ATP binding, identical protein binding, enzyme binding, protein homodimerization (Table 5). The results suggested that *I. chinensis* could exert their therapeutic effects by participating in the regulation of various biological processes (Figure 3).

**Table 3:** GO-BP analysis.

Signal pathway	*p-*value	Count
Positive regulation of transcription from RNA polymerase II promoter	3.57028 × 10^−7^	14
Signal transduction	2.82142 × 10^−5^	12
Response to xenobiotic stimulus	3.3837 × 10^−11^	11
Positive regulation of gene expression	6.72839 × 10^−7^	10
Positive regulation of transcription, DNA-templated	1.08731 × 10^−5^	10
Response to drug	1.04544 × 10^−7^	9
Negative regulation of transcription from RNA polymerase II promoter	5.99392 × 10^−4^	9
Protein phosphorylation	5.6852 × 10^−05^	8
Negative regulation of apoptotic process	7.72192 × 10^−05^	8
Peptidyl-serine phosphorylation	1.57615 × 10^−06^	7

**Table 4:** GO-CC analysis.

Signal pathway	*p*-value	Count
Cytoplasm	2.85031 × 10^−5^	24
Nucleoplasm	3.156 × 10^−5^	20
Plasma membrane	9.68707 × 10^−4^	20
Nucleus	7.52085 × 10^−3^	20
Cytosol	7.28669 × 10^−3^	19
Integral component of membrane	3.2415493 × 10^−2^	17
Macromolecular complex	2.7946 × 10^−10^	14
Membrane	6.305045 × 10^−3^	12
Endoplasmic reticulum membrane	1.24183 × 10^−4^	10
Mitochondrion	4.887025 × 10^−3^	9

**Table 5:** GO-MF analysis.

Signal pathway	*p-*value	Count
Protein binding	9.727706 × 10^−3^	34
ATP binding	1.22316 × 10^−6^	15
Identical protein binding	2.32923 × 10^−5^	14
Enzyme binding	2.54902 × 10^−10^	12
Protein homodimerization activity	1.36166 × 10^−06^	11
DNA binding	1.311848 × 10^−3^	10
Zinc ion binding	3.51011 × 10^−4^	9
Chromatin binding	4.26989 × 10^−5^	8
Protein kinase activity	1.17564 × 10^−4^	7
Protein serine/threonine kinase activity	1.41325 × 10^−4^	7

### 3.5 Analysis of KEGG enrichment pathway

KEGG is the main database for systematic analysis of gene function, genomic and proteomic information, where different proteins exercise their biological behaviors in coordination with each other, and it helps researchers to study protein and expression information as a whole network. Included in the database are illustrated cellular biochemical processes such as metabolism, membrane transport, signaling, cell cycle, and also information on homologous conserved subpathways (14).

**Figure 4:**
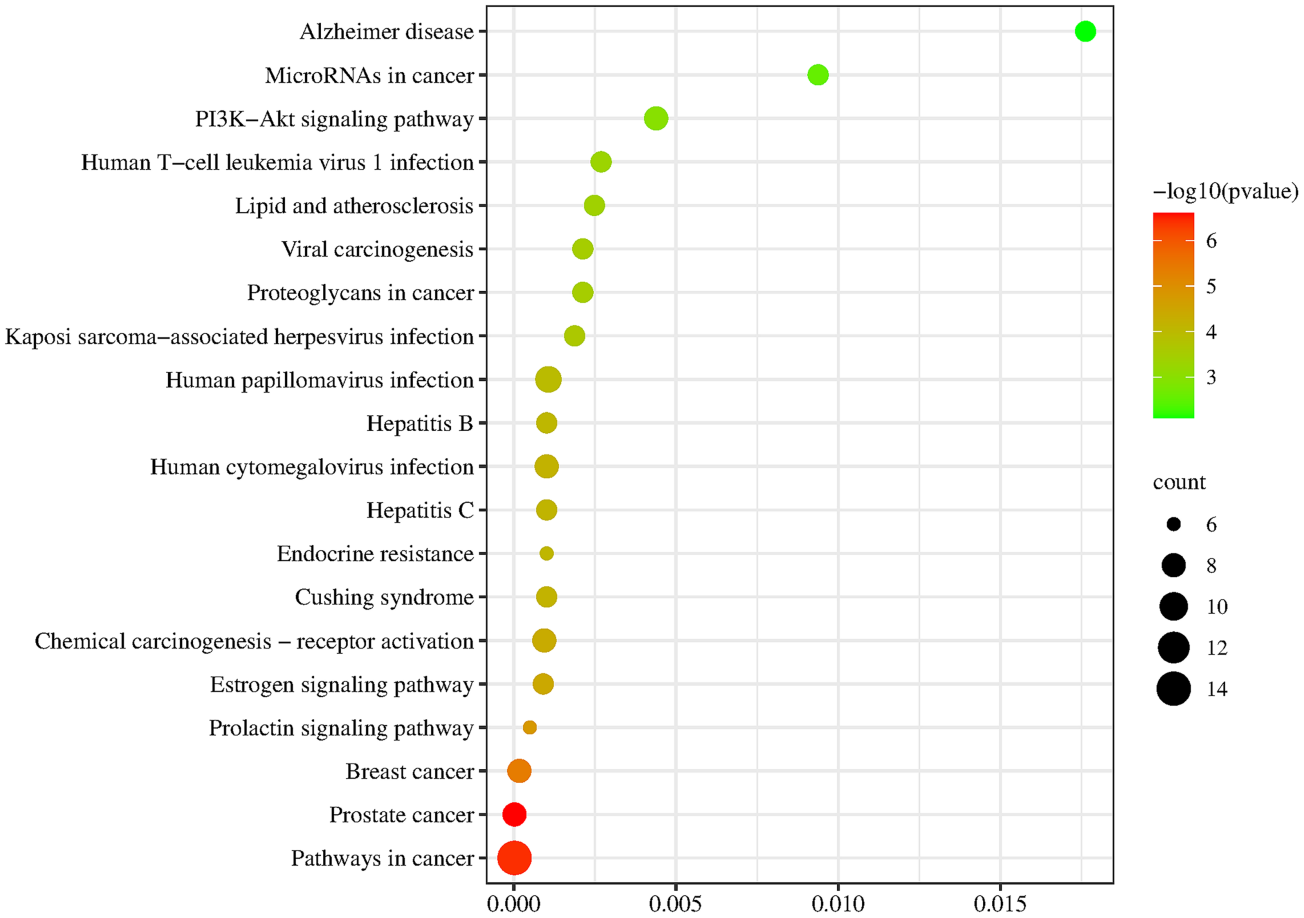
Enrichment results of key target protein KEGG. The larger the point was, the more relevant target points were; The redder the color, the higher the credibility.

KEGG analysis showed that the five most significantly enriched pathways were Pathways in cancer, Human papillomavirus infection, Prostate cancer, Breast cancer, Chemical carcinogenesis-receptor activation (Table 6 and Figure 4). Among the pathways associated with disease classes, the main ones are the Pathways in cancer, Human papillomavirus infection, Prostate cancer, Breast cancer, Chemical carcinogenesis – receptor activation, Human cytomegalovirus infection, Cushing syndrome, Hepatitis C, Hepatitis B, Kaposi sarcoma-associated herpesvirus infection, Viral carcinogenesis, Proteoglycans in cancer, Lipid and atherosclerosis, Human T-cell leukemia virus 1 infection, MicroRNAs in cancer, Alzheimer disease, etc. Among the pathways associated with signaling functions were PI3K-Akt signaling pathway, Estrogen signaling pathway, Prolactin signaling pathway, etc. It was suggested that the potential targets were involved in multiple signaling pathways acting in concert.

**Table 6:** Key target protein KEGG enrichment results.

Term	*p*-value	GENE
Pathways in cancer	3.85841 × 10^−7^	GSK3B, CREBBP, F2, PTGS2, ESR1, MMP9, EGFR, IL2, AR, CDK6, KIT, CDK2, AKT1, MAPK1
Human papillomavirus infection	1.12948 × 10^−4^	GSK3B, CREBBP, CDK6, CDK2, MAPK1, AKT1, PTGS2, TNF, EGFR
Prostate cancer	2.50934 × 10^−7^	GSK3B, AR, CREBBP, CDK2, MAPK1, AKT1, MMP9, EGFR
Breast cancer	4.20554 × 10^−6^	GSK3B, CDK6, KIT, MAPK1, AKT1, PGR, ESR1, EGFR
Chemical carcinogenesis - receptor activation	4.5876 × 10^−5^	AR, SRC, MAPK1, AKT1, PGR, AHR, ESR1, EGFR
Human cytomegalovirus infection	6.69942 × 10^−5^	GSK3B, CDK6, SRC, MAPK1, AKT1, PTGS2, TNF, EGFR
PI3K-Akt signaling pathway	1.070434 × 10^−3^	GSK3B, CDK6, KIT, CDK2, MAPK1, AKT1, EGFR, IL2
Estrogen signaling pathway	3.70105 × 10–^5^	SRC, MAPK1, AKT1, PGR, ESR1, MMP9, EGFR
Cushing syndrome	7.10189 × 10-^5^	GSK3B, CDK6, CDK2, MAPK1, AHR, EGFR, CYP17A1
Hepatitis C	7.62765 × 10^−5^	GSK3B, CDK6, CDK2, MAPK1, AKT1, TNF, EGFR
Hepatitis B	9.07914 × 10^−5^	CREBBP, SRC, CDK2, MAPK1, AKT1, TNF, MMP9
Kaposi sarcoma-associated herpesvirus infection	2.44053 × 10^−4^	GSK3B, CREBBP, CDK6, SRC, MAPK1, AKT1, PTGS2
Viral carcinogenesis	3.20198 × 10^−4^	CREBBP, CDK6, SRC, CHEK1, CDK2, CDK1, MAPK1
Proteoglycans in cancer	3.28737 × 10^−4^	SRC, MAPK1, AKT1, ESR1, TNF, MMP9, EGFR
Lipid and atherosclerosis	4.24369 × 10^−4^	GSK3B, CYP2C9, SRC, MAPK1, AKT1, TNF, MMP9
Human T-cell leukemia virus 1 infection	5.03384 × 10^−4^	CREBBP, CHEK1, CDK2, MAPK1, AKT1, TNF, IL2
MicroRNAs in cancer	2.82329 × 10^−3^	CREBBP, ABCB1, CDK6, MAPK1, PTGS2, MMP9, EGFR
Alzheimer disease	8.007899 × 10^−3^	GSK3B, APP, MME, MAPK1, AKT1, PTGS2, TNF
Prolactin signaling pathway	1.61592 × 10^−5^	GSK3B, SRC, MAPK1, AKT1, ESR1, CYP17A1
Endocrine resistance	8.24887 × 10^−5^	SRC, MAPK1, AKT1, ESR1, MMP9, EGFR

### 3.6 Analysis of KEGG signaling pathways related to cardiovascular and cerebrovascular

Cardiovascular and cerebrovascular diseases based on atherosclerosis are the main killers threatening human health. Years of research showed that the common pathological basis of cardiovascular and cerebrovascular diseases comes from atherosclerosis, so the key to prevent cardiovascular and cerebrovascular diseases is to prevent atherosclerosis (15).

There are many factors affecting cardiovascular and cerebrovascular diseases, such as vascular endothelial growth factor A (VEGF-A), a dimer glycoprotein encoded by VEGF-A gene, which plays a crucial role in the process of inducing vascular growth (16). Many studies have also confirmed that Akt and downstream molecular activity changes are related to some cardiovascular diseases. Minamino et al. (17) detected a vascular endothelial cell senescence phenotype in atheromatous plaque tissue. The findings suggested that statins improved endothelial function and reduced myocardial remodeling in the early stages of myocardial infarction through activation of Akt. Yang Hui et al. (18) prepared a hyperlipidemia mouse model, treated the water extract of *I. chinensis* and injected it intraperitoneally. The study showed that *I. chinensis* has an important contribution to improving the antioxidant capacity of hyperlipidemia mice, and could reduce the level of lipid peroxidation, but the specific deep mechanism needs further study.

Signaling pathways are important for the study of diseases, based on the key targets in *I. chinensis* and cardiovascular diseases, KEGG enrichment analysis showed that lipids and atherosclerosis was more significant signaling pathways. Therefore, taking this pathway as an example, it is of great significance to explore the role of the key target in the signal pathway of *I. chinensis*, and to explore the specific mechanism of disease and treat disease.

#### 3.6.1 Lipid and atherosclerosis signal pathway

The elevated level of low density lipoprotein (LDL) cholesterol is a major risk factor for atherosclerosis. LDL can accumulate in the blood vessel wall and be modified by oxidation. Oxidized LDL (oxLDL) leads to endothelial dysfunction, the expression of adhesion molecules and the recruitment of monocytes in the subendothelial space (19). The extracellular oxLDL binds to the membrane receptor Lox-1. LOX-1 mediates the recognition and internalization of oxLDL, activates the substrate ROCK2, which inhibits PI3K, and then generates PIP3, thereby activating AKT, which promotes Bclxl phosphorylation, thus inducing cell apoptosis (20, 21).

Advanced glycosylation end products (AGEs) are harmful compounds formed by the combination of protein or fat in the blood with sugar. They bind with the RAGE receptor on the cell membrane, and then activate and generate ROS (22). ROS indirectly activates Src, which in turn activates Rac1. Rac1 further activates MKK4/7, promotes JNK phosphorylation, and then promotes AP-1 phosphorylation. The phosphorylated AP-1 will enter the nucleus, regulate DNA transcription, and affect the expression of MCP-1, ICAM-1, and IL-8, thus affecting the attachment and activation of downstream monocytes. ERK activated in cytoplasm can indirectly activate NF-κB, NF-κB affects the expression of MMP1,3,9 and TNF-α by promoting DNA transcription, thus affecting damage, cell apoptosis and inflammation (23).

Minimally modified low density lipoprotein (mmLDL) is LDL in which only the lipid fraction is oxidized and the lysine residue in the Apo B100 structure is not destroyed (24). MmLDL binds with TLR2/4/6 receptor on cell membrane to activate ERK, which in turn indirectly activates rap1, and rap1 activates Rac, which in turn activates NADPH oxidase, promotes the expression of ROS *in vivo*, and indirectly promotes NF-κB. In addition, mmLDL promotes the separation of intracytoplasmic phosphorylated IκBα from NF-κB through a series of pathways, and the separated NF-κB promotes DNA transcription, thus affecting the expression of MMP1,3,9 and TNF-α, which have effects on monocyte attachment and activation, injury and apoptosis, and inflammatory response.

Tumor necrosis factor (TNF-α) is a cytokine involved in systemic inflammation, and also one of the many cytokines that cause acute response. Extracellular TNF-α binds to the TNF-R1 receptor on the cytomembrane, indirectly activating CASP8, and then activating CASP3, thereby affecting cell apoptosis and plaque instability (25).

Very low density lipoprotein(VLDL)is a kind of lipoprotein which is composed of apolipoprotein synthesized in the liver by using the intermediate metabolites of chyle granules, bile acid, fatty acid, sugar and protein. VLDL binds to VLDLR receptor on the cell membrane, activates substrate PI3K, connects with PI3K-AKT signal, promotes the expression of PIP3, and then activates AKT (26). AKT phosphorylates GSK3*β*, and the phosphorylated GSK3*β* phosphorylates NFAT, which promotes DNA transcription and has an effect on cell proliferation (27).

**Figure 5:**
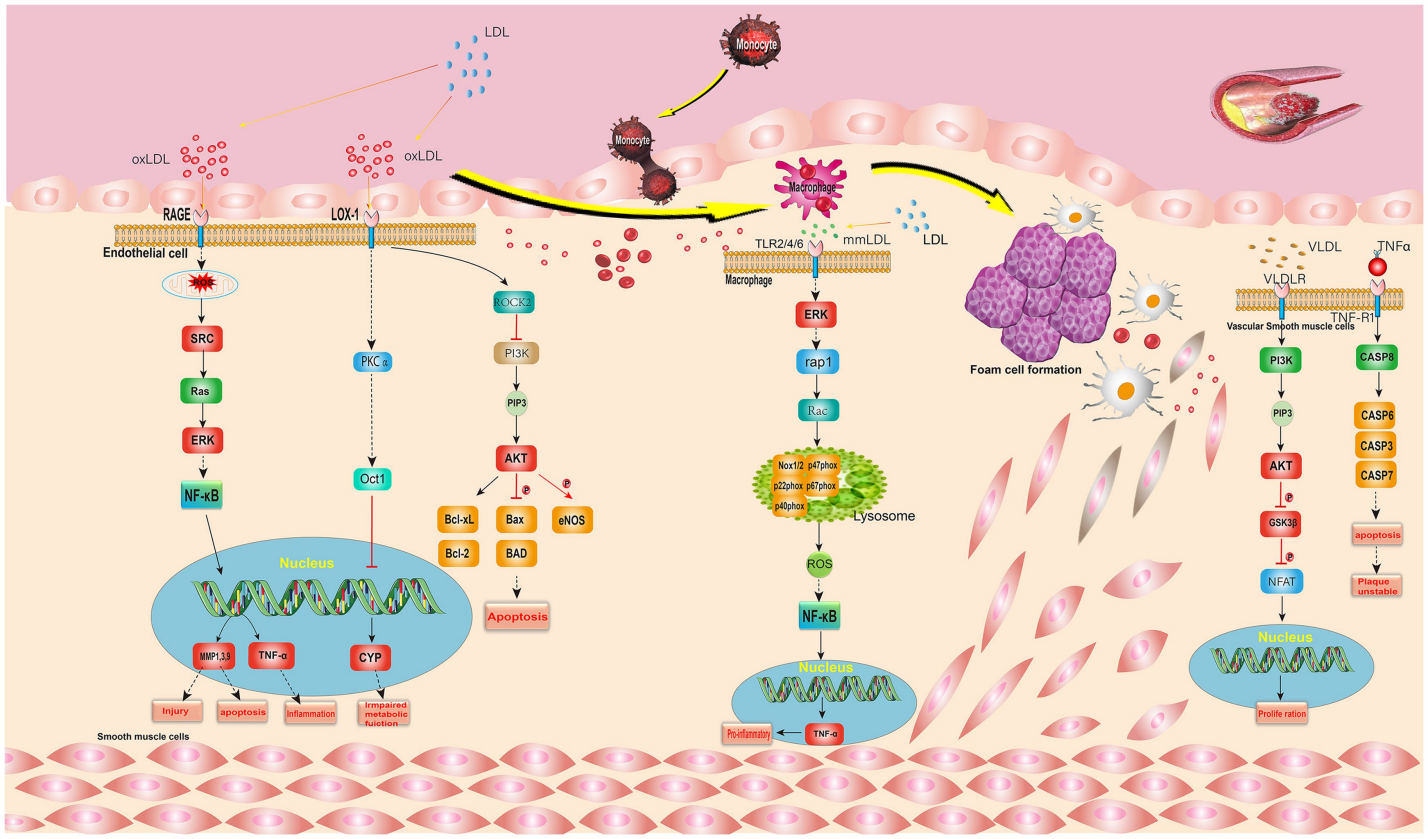
Lipid and atherosclerosis signal pathway. The gene marked red is the key target in *I. chinensis*.

The signal pathways are complex, and the proteins related to the key targets in the *I. chinensis* were all marked red (Figure 5), such as Src, TNFα, ERK, AKT, CYP, etc. It could be seen from the figure that most of the proteins in this pathway were distributed in the middle reaches, and they had important effects on inflammatory reaction, foam cell formation, cytokines, inflammatory signals, plaque instability, etc. The study of this pathway is helpful for anti atherosclerosis.

### 3.7 Analysis of related diseases enriched by targets

**Figure 6:**
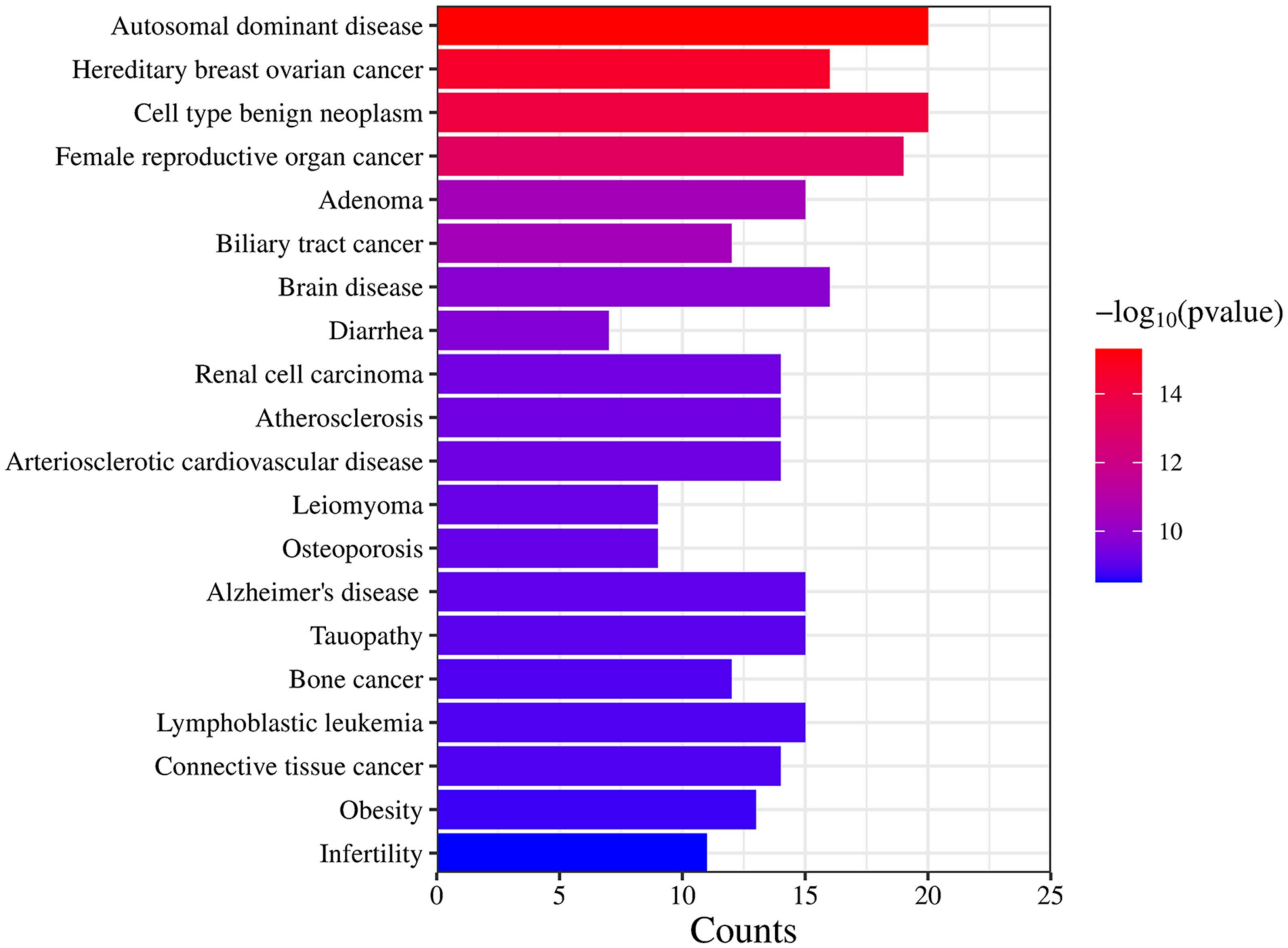
DO enrichment results for key target proteins. The longer the bar graph was, the more relevant targets were; The redder the color, the higher the credibility.

**Figure 7:**
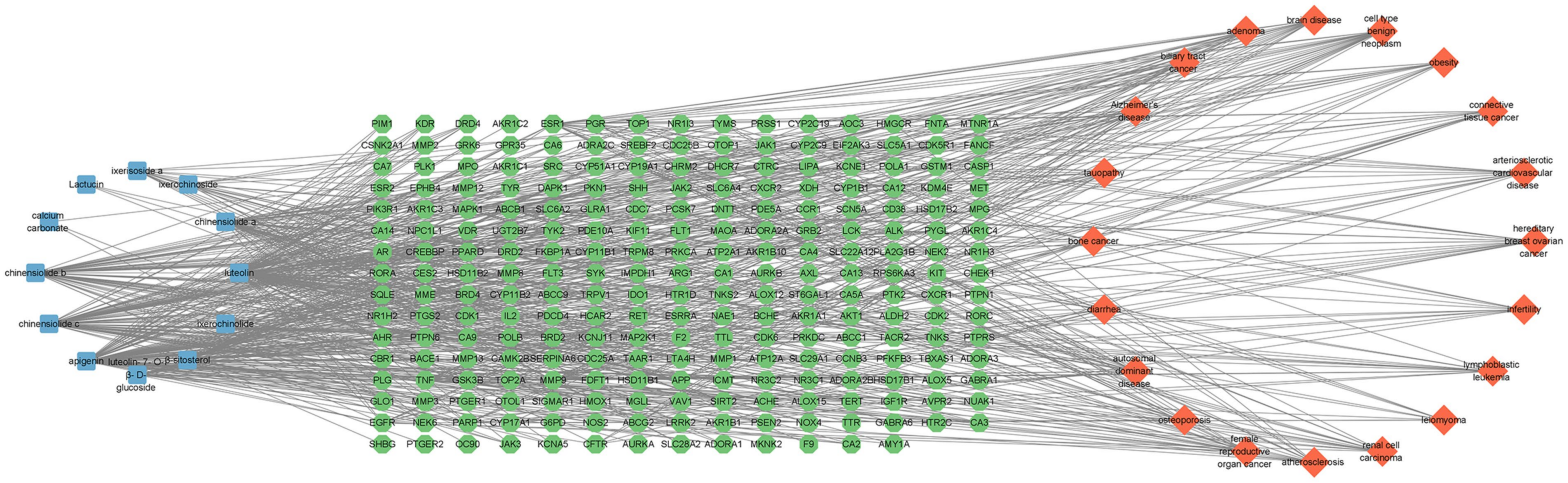
*Ixeris chinensis* component-disease-target map. In the figure, the left circle represented active chemical components, the middle square matrix represented target genes, and the right circle represented diseases.

DO data analysis was performed using Rstudio software, setting *p*-value <0.05 and Q value = 1. Disease enrichment analysis was performed on the key targets of *I. chinensis* action, removing the same diseases, and then extracting the 20 diseases with the highest number of targets and visualizing them using the Microbiology website (Figure 6 and Table 7). The main diseases included autosomal dominant disease, hereditary breast ovarian cancer, cell type benign neoplasm, female reproductive organ cancer, adenoma, biliary tract cancer, brain disease, diarrhea, renal cell carcinoma, atherosclerosis, arteriosclerotic cardiovascular disease, leiomyoma, osteoporosis, Alzheimer’s disease, tauopathy, bone cancer, connective tissue cancer, lymphoblastic leukemia, obesity, infertility, etc. The key targets proved to be highly relevant to these diseases. In order to explore the association between *I. chinensis* and diseases, Cytoscape 3.9.1 software was used to construct and visualize the “component-target-disease” network map using collected components, targets and disease data (Figure 7). The network consisted of 12 components, 238 targets and 20 diseases, which visually showed that multiple targets contained in one component could participate in the regulation of multiple diseases at the same time.

**Table 7:** DO enrichment results of key target protein.

Description	*p*-value	Gene
Autosomal dominant disease	4.97947 × 10^−16^	AKT1, TNF, EGFR, ESR1, SRC, MAPK1, AR, KIT, CDK2, GSK3B, PARP1, PGR, CYP19A1, ABCB1, CREBBP, CHEK1, AHR, TOP2A, CYP17A1, TOP1
Hereditary breast ovarian cancer	2.47998 × 10^−15^	AKT1, EGFR, ESR1, MAPK1, AR, CDK2, PARP1, PGR, CYP19A1, ABCB1, CREBBP, CHEK1, AHR, TOP2A, CYP17A1, TOP1
Cell type benign neoplasm	8.98661 × 10^−15^	AKT1, TNF, EGFR, ESR1, SRC, PTGS2, MAPK1, MMP9, AR, PARP1, PGR, CYP19A1, ABCB1, AHR, TOP2A, CYP17A1, DRD2, MME, CA9, HSD11B1
Female reproductive organ cancer	6.49841 × 10^−14^	AKT1, TNF, EGFR, ESR1, SRC, PTGS2, MAPK1, MMP9, AR, KIT, CDK1, PGR, CYP19A1, ABCB1, ABCG2, TYMS, TOP2A, MME, CA9
Adenoma	2.96803 × 10^−11^	AKT1, EGFR, ESR1, PTGS2, MAPK1, MMP9, AR, PGR, ABCB1, AHR, TOP2A, DRD2, MME, CA9, HSD11B1
Biliary tract cancer	3.26756 × 10^−11^	AKT1, TNF, EGFR, ESR1, PTGS2, MAPK1, MMP9, ABCB1, ABCG2, TOP2A, CYP2C19, MME
Brain disease	1.5651 × 10^−10^	TNF, EGFR, ESR1, PTGS2, MMP9, IL2, AR, APP, PGR, ABCB1, ABCG2, CYP2C9, SLC6A4, CYP2C19, DRD2, HSD11B1
Diarrhea	2.17837 × 10^−10^	TNF, EGFR, PTGS2, ABCB1, ABCG2, SLC6A4, CFTR
Renal cell carcinoma	4.37165 × 10^−10^	AKT1, TNF, EGFR, ESR1, PTGS2, MAPK1, MMP9, IL2, AR, KIT, ABCB1, TYMS, MME, CA9
Atherosclerosis	4.72123 × 10^−10^	AKT1, TNF, ESR1, PTGS2, MMP9, AR, CYP19A1, AHR, CYP2C9, F2, SLC6A4, CYP2C19, AKR1B1, HMGCR
Arteriosclerotic cardiovascular disease	4.90545 × 10^−10^	AKT1, TNF, ESR1, PTGS2, MMP9, AR, CYP19A1, AHR, CYP2C9, F2, SLC6A4, CYP2C19, AKR1B1, HMGCR
Leiomyoma	6.40375 × 10^−10^	TNF, EGFR, ESR1, AR, PARP1, PGR, CYP19A1, CYP17A1, DRD2
Osteoporosis	7.04139 × 10^−10^	TNF, ESR1, MAPK1, AR, KIT, CYP19A1, ABCB1, CYP17A1, HSD11B1
Alzheimer’s disease	8.15987 × 10^−10^	AKT1, TNF, ESR1, PTGS2, MAPK1, GSK3B, APP, PARP1, CDK1, CYP19A1, ABCB1, F2, SLC6A4, MME, CFTR
Tauopathy	9.28254 × 10^−10^	AKT1, TNF, ESR1, PTGS2, MAPK1, GSK3B, APP, PARP1, CDK1, CYP19A1, ABCB1, F2, SLC6A4, MME, CFTR
Bone cancer	1.22472 × 10^−9^	AKT1, TNF, EGFR, ESR1, PTGS2, MAPK1, MMP9, AR, KIT, ABCB1, CHEK1, TOP1
Connective tissue cancer	1.22959 × 10^−9^	AKT1, TNF, EGFR, ESR1, SRC, PTGS2, MAPK1, MMP9, AR, KIT, ABCB1, CHEK1, TOP2A, TOP1
Lymphoblastic leukemia	1.23466 × 10^−9^	AKT1, TNF, PTGS2, MAPK1, MMP9, IL2, GSK3B, PARP1, CDK6, ABCB1, ABCG2, AHR, TYMS, TOP2A, MME
Obesity	1.80466 × 10^−9^	TNF, ESR1, MAPK1, MMP9, AR, APP, PGR, CYP19A1, ABCB1, NR3C1, SLC6A4, DRD2, HSD11B1
Infertility	3.01154 × 10^−9^	TNF, ESR1, MMP9, AR, PGR, CYP19A1, ABCB1, AHR, F2, CYP17A1, CFTR

### 3.8 Molecular docking technique to predict the binding ability of the active components and key targets of *I. chinensis*

**Figure 8:**
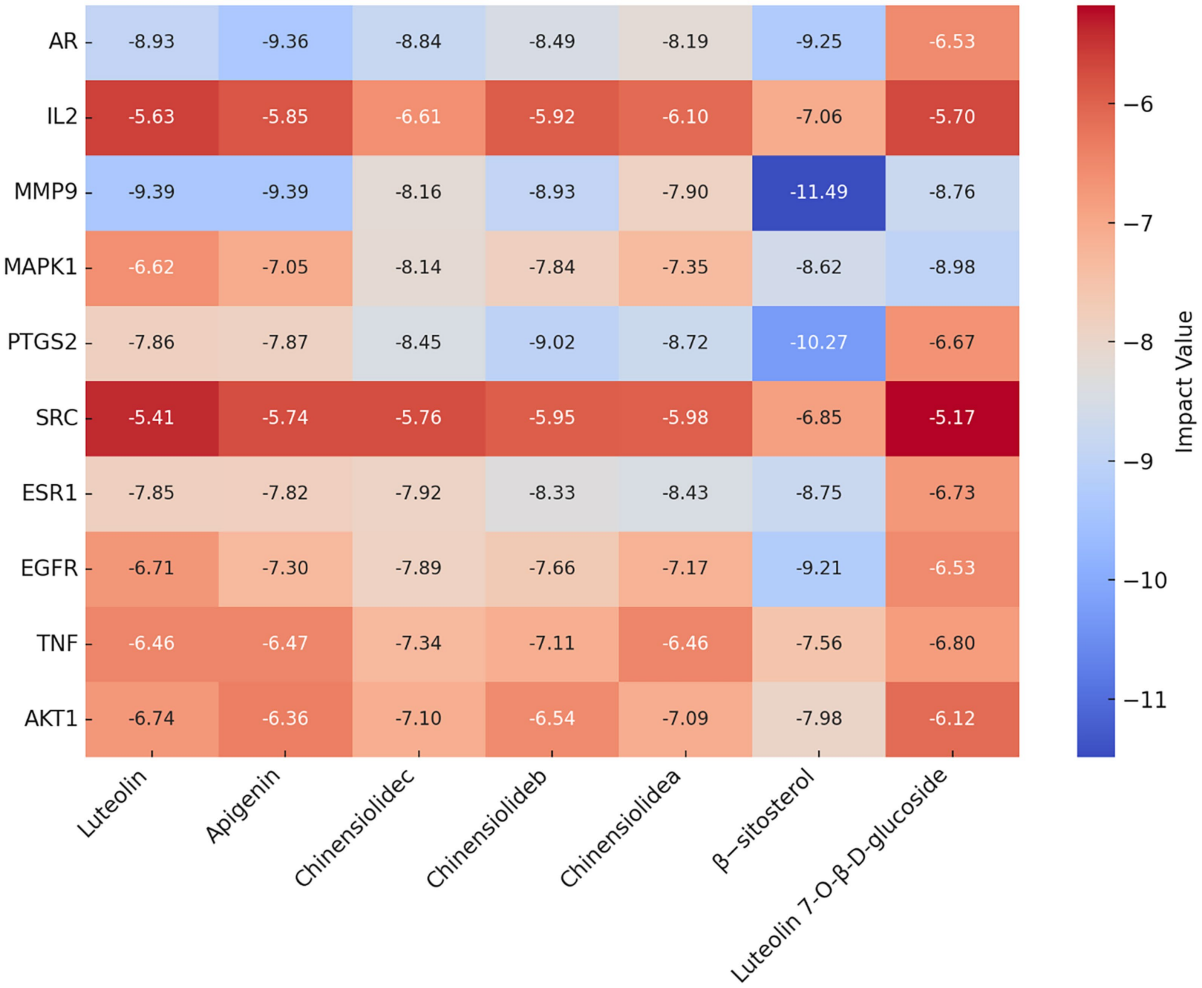
Docking and binding energy information of active ingredients and key target molecules in *Ixeris chinensis*. The redder the color, the better the binding ability.

**Figure 9:**
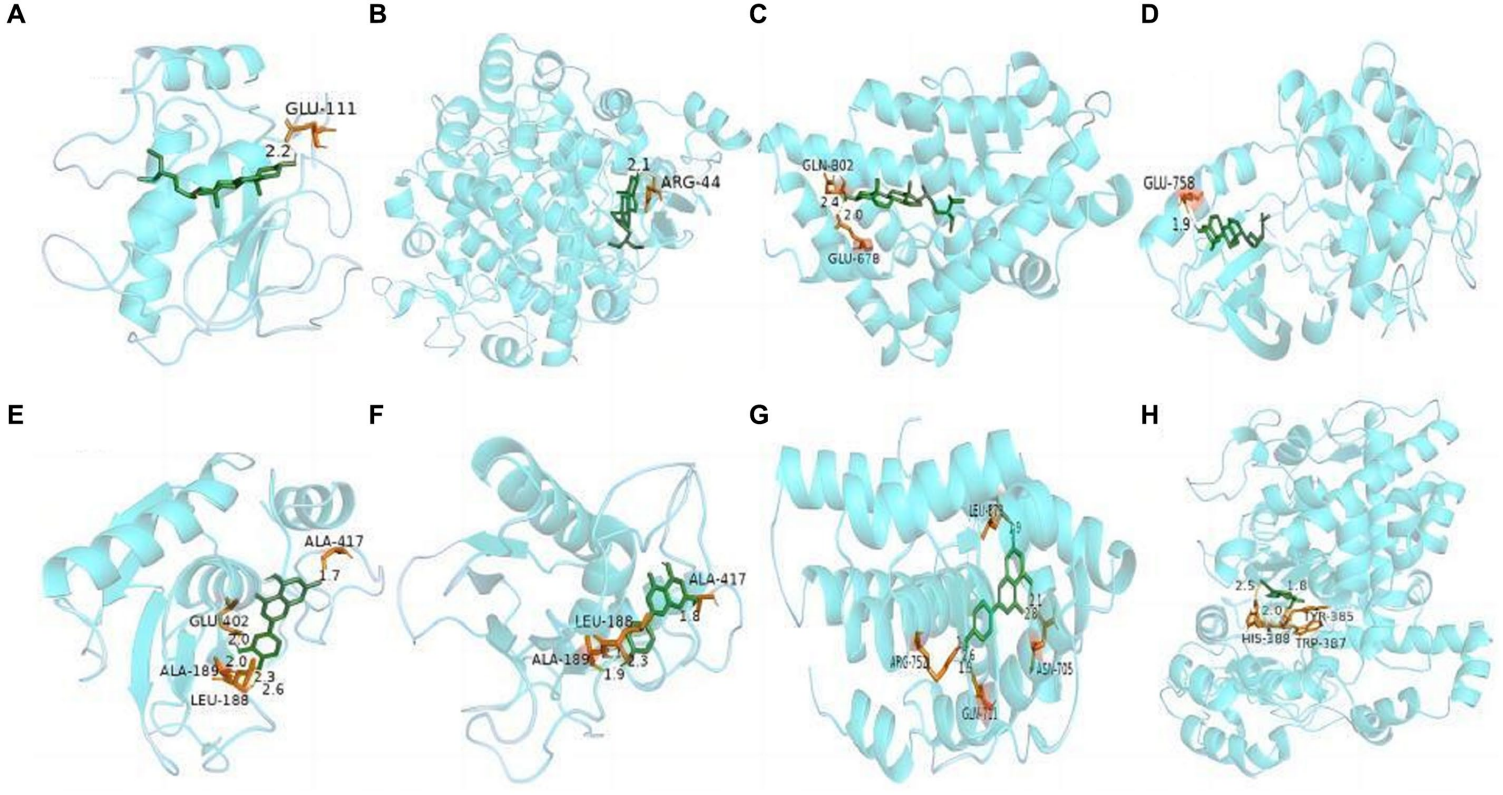
Docking analysis of the active components and key targets of *I. chinensis*. In the figure, the blue macromolecule is the target protein, and the green smaller molecule is the active component. **(A)** *β*-sitosterol and MMP9, **(B)** *β*-sitosterol and PTGS2, **(C)** *β*-sitosterol and AR, **(D)** *β*-sitosterol and EGFR, **(E)** luteolin and MMP9, **(F)** apigenin and MMP9, **(G)** apigenin and AR, **(H)** chinensiolide b and PTGS2.

Molecular docking technology is of great significance for further research in plant chemistry and biology (28). The current study docked each of the 7 active ingredients in *I. chinensis* with 10 key targets, yielding 70 docking results (Figure 8). The results revealed that luteolin, apigenin, chinensiolide c, chinensiolide b, chinensiolide a, *β*-sitosterol, and luteolin-7-*O*-*β*-D-glucoside had better docking ability with EGFR, ESR1, PTGS2, MAPK1, MMP9, and AR. The lower the binding energy, the better the molecule’s ability to bind to the protein (Table 8). Among them, MMP9 showed strong docking ability with each active ingredient, and *β*-sitosterol also showed strong binding ability with each protein. The docking results of the top 8 components with strong binding activity (binding energy ≤ −7 kcal·mol-1) to the targets were visualized and presented using PyMOL software (Figure 9). *β*-sitosterol forms one hydrogen bond with MMP9 via amino acid residue GLU-111, one hydrogen bond with PTGS2 via amino acid residue ARG-44, two hydrogen bonds with AR via amino acid residues GLN-802 and GLU-687, and one hydrogen bond with EGFR via amino acid residue GLU-758 forming one hydrogen bond; luteolin forming four hydrogen bonds with MMP9 via amino acid residues ALA-189, ALA-417, GLU-402, and LEU-188; apigenin forming three hydrogen bonds with MMP9 via amino acid residues ALA-189, ALA-417, and LEU-188, and three hydrogen bonds with MMP9 via amino acid residues ARG-752, ASN-705, GLN-711, and LEU-873 to form four hydrogen bonds with AR, and chinensiolide b to form three hydrogen bonds with PTGS2 via amino acid residues HIS-388, TRP-387, and TYR-385. The high binding energy between the main active components and key targets in *I. chinensis* indicated that *I. chinensis* might play its therapeutic role by regulating the above related targets.

**Table 8:** The binding ability of the active components and key targets of *Ixeris chinensis.*

**Gene**	**Luteolin**	**Apigenin**	**Chinensiolide c**	**Chinensiolide b**	**Chinensiolide a**	**β-sitosterol**	**Luteolin 7-*O*-*β*-D-glucoside**
AKT1	−6.74	−6.36	−7.1	−6.54	−7.09	−7.98	−6.12
TNF	−6.46	−6.47	−7.34	−7.11	−6.46	−7.24	−6.8
EGFR	−6.71	−7.3	−7.89	−7.66	−7.17	−9.21	−6.53
ESR1	−7.85	−7.82	−7.92	−8.33	−8.43	−8.75	−6.73
SRC	−5.41	−5.74	−5.76	−5.95	−5.98	−6.85	−5.17
PTGS2	−7.86	−7.87	−8.45	−9.02	−8.72	−10.27	−6.67
MAPK1	−6.62	−7.05	−8.14	−7.84	−7.35	−8.62	−8.98
MMP9	−9.39	−9.39	−8.16	−8.93	−7.9	−11.49	−8.76
IL2	−5.63	−5.85	−6.61	−5.92	−6.1	−7.06	−5.7
AR	8.93	−9.36	−8.84	−8.49	−8.19	−9.25	−6.53

### 3.9 Meta analysis results

#### 3.9.1 Literature search and screening results

**Figure 10:**
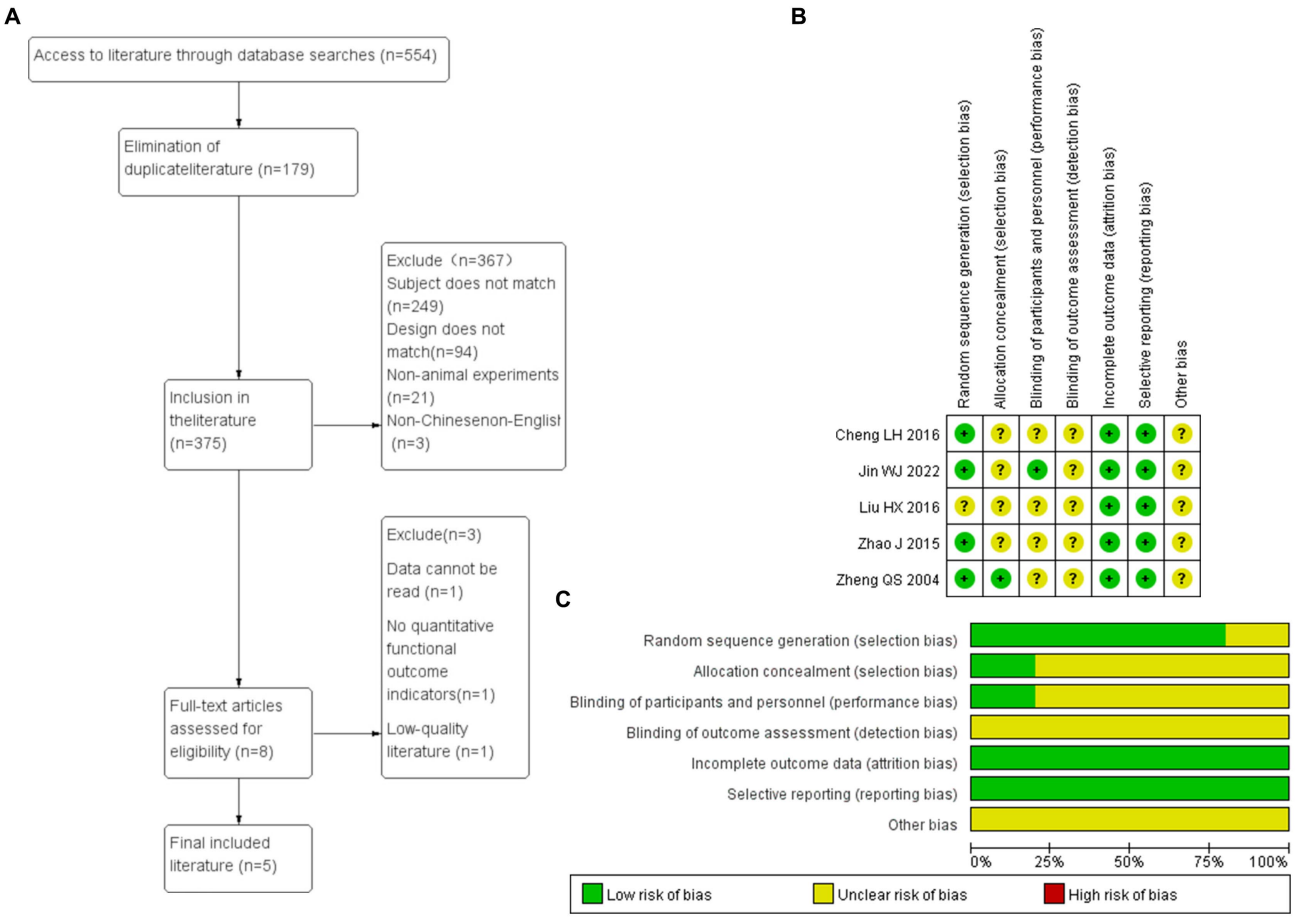
**(A)** Flow chart of document screening. **(B,C)** Risk assessment of literature bias. Green is low risk bias, yellow is unknown risk bias, and red is high risk bias. The length of the bar indicates the proportion of this bias in the total number of articles.

The document screening process and basic characteristics of included documents are shown in the figure below (Figure 10 and Table 8). A total of five randomized controlled trials (RCTs) were conducted, including studies employing Sprague–Dawley (SD) rats, ICR mice, C57BL/6J mice, Wistar rats, and KM mice. For modeling, one study was fed with high-fat and high-sugar diets, two studies used carbon tetrachloride (CCl4), and two studies were fed with high-fat diets. The interventions were mainly treated with *I. chinensis* water extract. The diseases included in the studies mainly consisted of two cases of lipid-based diseases and three cases of hepatitis-based diseases. The included outcome indicators contained TC, TG, HDL-C, LDL-C, and MDA.

#### 3.9.2 Document quality evaluation

The five research baselines included were comparable. In the Random sequence generation, four studies described the random allocation method in detail and were rated as low risk bias, while one study only mentioned the word “random” without specifying the specific method and was rated as unknown risk bias. In the Allocation consideration, only one described the allocation concealment scheme, which was rated as low risk bias, and the rest as unknown risk bias. Among Blinding, only one described the allocation concealment scheme, which was rated as low risk bias, and the rest as unknown risk bias. In the Incomplete outcome data, all studies reported complete results and were rated as low risk bias; In selective reporting, none of the five studies reported selectively, which was rated as low risk bias. In Other bias, five studies were unable to judge whether there were other sources of bias, and all were rated as unknown risk bias (Figure 10).

#### 3.9.3 Statistical analysis results

**Figure 11:**
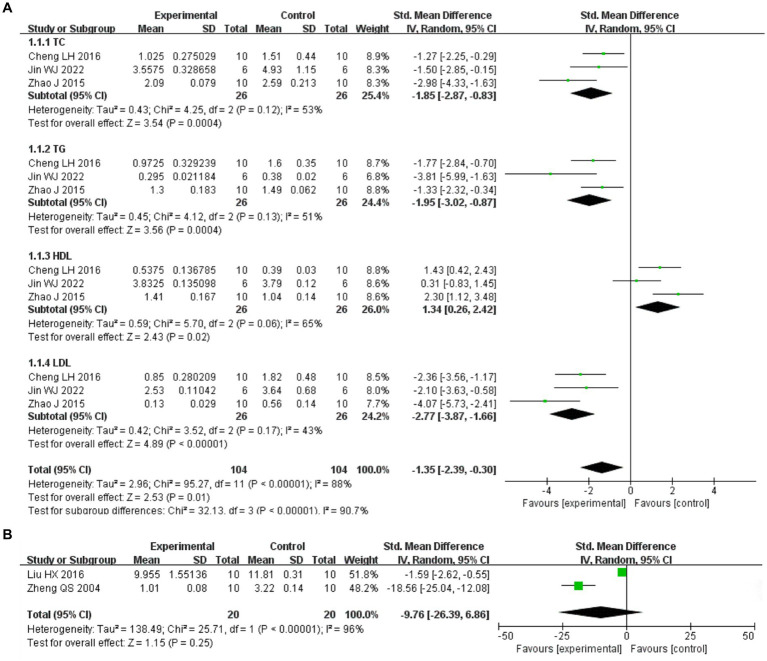
The study was grouped according to outcome indicators. The random effect model was used for analysis. Heterogeneity was calculated using I2. The summary estimates of the standard mean difference and 95% confidence zone were calculated using Review Manager 5.4.1. **(A)** Take the four items of blood lipid as the outcome index. **(B)** Take MDA as the outcome indicator.

Included in the study, three articles reported four changes in blood lipids (29–31) (Figure 11A). The above three articles were divided into four subgroups according to blood lipid indicators. Heterogeneity between studies (*I*^2^ > 50%) was analyzed by random effect model. Meta analysis results showed that *I. chinensis* could significantly reduce the blood TC level, [SMD = –1.85, 95% CI (−2.87, −0.38), *I*^2^ = 53%, *p* = 0.0004], indicating significant heterogeneity and statistically significant difference. *I. chinensis* could also significantly reduce the blood TG level, [SMD = –1.95, 95% CI (−3.02, −0.87), I2 = 51%, *p* = 0.0004], indicating significant heterogeneity and statistically significant difference. *I. chinensis* showed no statistically significant difference in improving HDL-C [SMD = 1.34, 95% CI (0.26, 2.42), *I*^2^ = 65%, *p* = 0.02]. *I. chinensis* significantly reduced blood LDL-C levels, [SMD = –2.77, 95% CI (−3.87, −1.66), *I*^2^ = 43%, *p* < 0.00001], suggesting that the difference was statistically significant. The sensitivity analysis of the included literatures was carried out one by one with the method of exclusion. None of the literatures had a great impact on the results of this Meta-analysis, which meant that this study has a good stability.

Among the included studies, two articles reported changes in MDA with heterogeneity between studies (*I*^2^ > 50%), analyzed using a random-effects model (32, 33) (Figure 11B). The difference in *I. chinensis* in improving HDL-C was not statistically significant [SMD = −9.76, 95% CI (−26.39, 6.86), *I*^2^ = 96%, *p* = 0.25].

#### 3.9.4 Publication bias analysis

**Figure 12:**
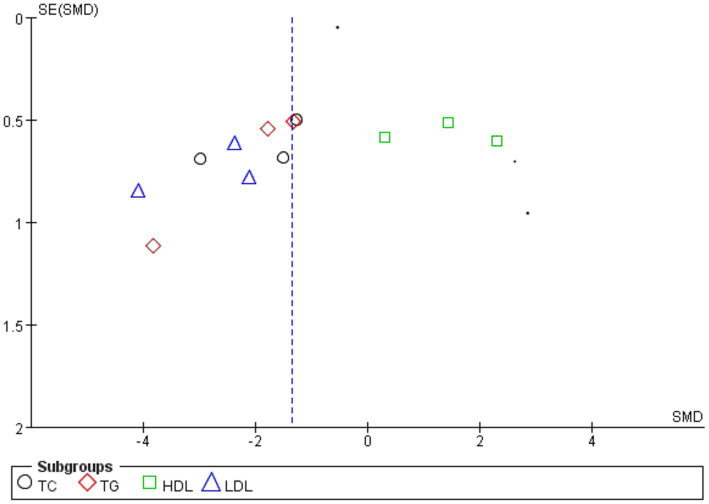
Funnel chart analysis of publication bias with four blood lipids as outcome indicators.

We used the four indicators of blood lipids as an example to create a funnel plot. Funnel diagram showed that the included articles were asymmetric with the symmetry axis as the boundary, indicating that there was publication bias, which might be related to the small sample size of the included articles (Figure 12).

## 4 Discussion

As a traditional Chinese herbal medicine, *I. chinensis* has complex types and various functions. It has been used as a medicinal material for lowering blood glucose, cholesterol, diuresis, bloodpressure, anticoagulation, liver protection, antiinflammatory, antibacterial, anti-tumor and so on (6). The emergence of traditional.

Chinese medicine network pharmacology provides an opportunity to systematically explore the molecular complexity of traditional Chinese medicine prescriptions and the molecular relationship between traditional Chinese medicine ingredients and complex diseases (34). In recent years, the pharmacological effects of *I. chinensis* have been widely studied, but the specific mechanism of disease action is still unclear and needs to be further studied. Therefore, we should start from the analysis of active components to explore the key role targets of *I. chinensis*, and carry out GO and KEGG analysis, conduct molecular docking which is of great significance to predict the biological process, cell composition, molecular function and signal pathway of *I. chinensis*, and has a profound influence on the research of specific disease mechanism and the development of new drugs.

As a traditional Chinese herbal medicine, there are many kinds of *I. chinensis*. Now we pay more attention to the pharmacodynamic material basis of *I. chinensis*, that is, the effective components in the curative effect, which requires us to screen and analyze the effective components in it. By searching “*I. chinensis* “in HERB database, the current study found 12 active ingredients of it, including apigenin, *β*-sitosterol, luteolin, luteolin-7-*O*-*β*-D-glucoside and so on. These active ingredients were used as the basis of the whole analysis of *I. chinensis*.

The active compounds of *I. chinensis* have many targets. The active compounds of *I. chinensis* were input into swiss target prediction for target gene prediction. The targets without corresponding gene names were deleted by Uniprot database, and the related targets were screened out. To explore the relationship between the active compounds of *I. chinensis* and the target sites, Cytoscape3.9.1 was used to construct the *I. chinensis* composition-target network diagram. There were 238 targets of *I. chinensis*. Two hundred and thirty-eight potential targets were input into STRING database for protein interaction analysis, and free targets were eliminated. Cytoscape 3.9.1 software was used to construct PPI network, and 40 key targets were screened out. They were mainly AKT1, TNF, EGFR, ESR1, SRC, PTGS2, MAPK1, which were regarded as important proteins of *I. chinensis*. It was suggested that it might play a therapeutic role in diseases through the interaction of active compounds with key targets. Most of the key targets were closely related to monocyte attachment and activation, injury and apoptosis, inflammation, matrix digestion, cell migration, differentiation, proliferation and other biological functions. They had similar biological effects and synergistic effects of multiple targets. Akt1 is an important gene regulating cell survival and proliferation, which can regulate tumor proliferation, metastasis and invasion. Transplanting mesenchymal stem cells overexpressing Akt1 into rat myocardium can reduce infarct size and delay cardiac remodeling (35). TNF is a substance that can damage tumor cells and make them necrotic. TNF-α plays a role in inducing cell apoptosis, regulating the immune response of the body, regulating the vascular system of tumor tissue, and inducing programmed cell necrosis. It plays different regulatory roles in many malignant tumors, such as gastric cancer, liver cancer, breast cancer, etc. (36). EGFR belongs to the epidermal growth factor receptor family, which is activated after binding with ligands. It transmits information through Ras/Raf/MEK/ERK/MAPK pathway, PI3K/AKT (PKB) pathway and JAK/STAT pathway, thus affecting tumor cell proliferation, angiogenesis, invasion and metastasis (37). Estrogen Receptor 1 (ESR1) expression is lost or diminished in human Hepatocellular carcinoma (HCC) cells and liver tumors, suggesting a potential protective role of estrogen signaling in HCC and that low ESR1 gene expression plays.

an important role in the development of hepatocellular carcinoma (38). The sparse representation based classifier gene is the earliest proto oncogene discovered by human beings. Its protein product, Src, is the most widely distributed protein kinase *in vivo*. It participates in a series of physiological activities such as regulating cell proliferation and differentiation by phosphorylating various signal molecules in cells (39). PTGS2 gene is an inducible immediate response gene, and its expression is rapidly upregulated by certain cytokines, growth factors, inflammatory mediators, pro-oncogenic factors and other stimulating factors during the occurrence of pathological responses such as inflammation or tumor, and its main product catalyzed by PG is an important inflammatory mediator in liver injury, which is an important link in inflammation (40). Mitogen-activated protein Kinase (MAPK) signal pathway is an important inflammation related signal pathway. Some studies have shown that MAPK1 has neuroprotective effect after stroke, and some studies have shown that MAPK1 has harmful effects on stroke due to its activation promoting inflammation and oxidative stress and inhibiting and reducing ischemic injury (41).

Comprehensive analysis of the results of “component-target” network and “component-target-disease network,” The key components in *I. chinensis* were apigenin, chinensiolide c, chinensiolide a, chinensiolide b, luteolin, luteolin-7-*O*-*β*-D-glucoside and *β*-sitosterol, which were dominant in the network. According to relevant literature reports, apigenin, luteolin, luteolin-7-*O*-*β*-D-glucoside belong to flavonoids (6, 42–44), chinensiolide c, chinensiolide a and chinensiolide b belong to sesquiterpenes (45)，*β*-sitosterol belongs to triterpenes and steroids (6). Flavonoids have the advantages of scavenging free radicals, protecting liver, protecting heart and brain, regulating blood lipids and so on (46). Sesquiterpenes have a wide range of biological activities, such as anti-tumor, antibacterial, anti-inflammatory, anti-neurotoxic, antiviral, immunosuppressive, hepatoprotective and heart-strengthening activities (47). Triterpenes and steroids have anti-inflammatory, antibacterial and anti-tumor effects (48). Studies have shown that apigenin can inhibit the proliferation of colorectal cancer CL187 cells and promote apoptosis by inhibiting PI3K/Akt signaling pathway and regulating the expression of MAPK signaling pathway related proteins (49). Apigenin can inhibit the EMT of liver tissue cells by inhibiting PDK1/AKT signal pathway through liver bypass, and play an anti-fibrosis role (50). Luteolin can eliminate free radicals, enhance the activity of antioxidant enzymes, regulate proinflammatory mediators, and inhibit IκB kinase *β* Phosphorylation of subunits, down regulating TNF-α and the mRNA level of IL-6 (51). Luteolin-7-*O*-*β*-D-glucoside has protective effect on myocardial cells cultured under ischemia and hypoxia. Its mechanism may be related to scavenging oxygen free radicals, stabilizing cell membrane and inhibiting apoptosis (52). The water extract of *I. chinensis* A can effectively inhibit the growth of lung adenocarcinoma A549 cells, liver cancer Blx10-7402 cells and LoVo cells *in vitro*, and has strong anti-tumor activity (53). The sesquiterpene lactones, chinensiolide A-C, show strong anti-inflammatory and anti-tumor activities (54, 55). The pharmacological studies of the above active ingredients were in accordance with the results of *I. chinensis* network analysis, which was speculated to be an important component of *I. chinensis* to exert drug effects, and to a certain extent, it provided the value of medical research for the treatment of diseases.

Molecular docking technology can analyze the optimal binding sites between active components and targets, providing valuable insights into the mechanisms of drug action in treating diseases. Lin Shenghua and others (56) conducted molecular docking on the top 6 targets and the top 3 active components of Xuefu Zhuyu oral liquid for anti-thrombotic activity. The binding energies were between −5 and − 9.5KJ/mol, indicating that the active components of Xuefu Zhuyu oral liquid effectively bind with disease-related targets. Through molecular docking technology, the docking results between the main active components of 7 kinds of *I. chinensis* found in this study and key targets were pretty good, with binding energies concentrated between −7 and -9KJ/mol, indicating that the main active components of *I. chinensis* and key targets have good affinity, providing a basis for future drug design.

GO and KEGG analysis revealed that the biological processes of *I. chinensis* were mainly enriched in positive regulation of transcription from RNA polymerase II promoter, signal transduction, response to xenobiotic stimulus, positive regulation of gene expression, positive regulation of transcription, DNA-templated, response to drug, negative regulation of transcription from RNA polymerase II promoter, protein phosphorylation, negative regulation of apoptotic process, peptidyl-serine phosphorylation, etc. The potential action pathways of *I. chinensis* included 103 pathways of Pathways in cancer, Chemical carcinogenesis-receptor activation, PI3K-Akt signaling pathway, Hepatitis B, Proteoglycans in cancer, Lipid and atherosclerosis, etc. AKT1, TNF, EGFR and other proteins that regulated tumor cell proliferation, migration, apoptosis, inflammatory expression, immune response, MAPK1, MMP9, ESR1 and other proteins participated in nerve cell regulation, brain tissue repair, and regulation of vascular function were also at the center of the PPI network, indicating that *I. chinensis* may play a role in promoting tumor cell death, regulating blood lipids, and protecting the heart and brain by regulating these targets. In addition, the active compounds of *I. chinensis* could regulate inflammatory pathways, and the pathways with a high degree of significance include TNF signaling pathway, PI3K/AKT signaling pathway, and Estrogen signaling pathway, and the inflammatory response was related to the occurrence and development of diseases. Tumor necrosis factor-α (TNF-α) is a cytokine involved in systemic inflammation and is also among the many cytokines that cause acute phase responses. Extracellular TNF- α bound to the TNF-R1 receptor on the cytosolic membrane and indirectly activates CASP8, which in turn activated CASP3, and thus had an impact on apoptosis and plaque instability (57, 58). In addition, lignans and lignans 7-*O*-*β*-D-glucoside scavenged free radicals and enhanced antioxidant enzyme activity. It was evident that the active compounds of *I. chinensis* cought have myocardial protective and antiatherosclerotic effects by modulating the above-mentioned inflammatory pathways (59).

The DO analysis found that, *I. chinensis* could be applied to tumors such as hereditary breast, ovarian cancer, cell type benign neoplasm, female reproductive organ cancer, adenoma, biliary tract cancer, renal cell carcinoma, bone cancer, connective tissue cancer. It could also be applied to diseases such as autosomal dominant disease, brain disease, diarrhea, atherosclerosis, arteriosclerotic cardiovascular disease, leiomyoma, osteoporosis, Alzheimer’s, tauopathy, lymphoblastic leukemia, obesity, infertility, etc. It could be seen that the *I. chinensis* could be mainly used to treat cancer related diseases, visceral system diseases, reproductive system diseases, nervous system diseases, musculoskeletal system diseases, lymphatic system diseases, etc.

In conclusion, network pharmacology research can shorten the research process of molecular targets (60). Researchers can use network pharmacology studies to help discover effective and specific compounds for treating diseases (61). Based on meta analysis, network pharmacology and molecular docking methods, this study preliminarily explored the active components, key targets, docking binding energy, signal pathways and disease prediction of *I. chinensis*. It further verified the feasibility of *I. chinensis* as a drug to treat many diseases, and provided a basis for exploring the specific mechanism in treating diseases and developing new drugs. At the same time, current study found that in the five articles included, the extract of *I. chinensis* had a good effect on the improvement of blood lipid indicators. However, due to the small sample size, general literature quality, and fewer types of diseases studied, the reliability of outcomes had been affected to some extent, so more *in vitro* and *in vivo* experiments are needed to verify in the future.

## Author contributions

ZN: Resources, Writing – original draft. ZM: Writing – original draft, Conceptualization. XQ: Data curation, Writing – original draft. YG: Formal analysis, Software, Writing – review & editing. CR: Methodology, Project administration, Writing – original draft, Writing – review & editing. YW: Resources, Writing – original draft. YY: Investigation, Writing – original draft.

## Footnotes

^1^ https://old.tcmsp-e.com/tcmsp.php

^2^ http://herb.ac.cn/Search/

^3^ https://pubchem.ncbi.nlm.nih.gov/

^4^ http://swisstargetprediction.ch/

^5^ https://www.uniprot.org/

^6^ https://cn.string-db.org/

^7^ https://david.ncifcrf.gov/

^8^ https://pubmed.ncbi.nlm.nih.gov/

^9^ http://isiknowledge.com/

^10^ https://www.cnki.net/

^11^ http://www.wanfangdata.com.cn/
